# Stabilizing the unstable: Tuberculosis of the odontoid process with atlanto‐occipital instability—Case report and review of literature

**DOI:** 10.1002/ccr3.8379

**Published:** 2023-12-28

**Authors:** Shyam Duvuru, Vivek Sanker, Tirth Dave, Pratik Agarwal, Naureen Syed

**Affiliations:** ^1^ Consultant Neurosurgeon Apollo Specialty Hospitals Madurai Tamil Nadu India; ^2^ Team Erevnites Trivandrum India; ^3^ Noorul Islam Institute of Medical Sciences Trivandrum India; ^4^ Bukovinian State Medical University Chernivtsi Ukraine; ^5^ Lokmanya Tilak Municipal Medical College Mumbai India; ^6^ UT MD Anderson Cancer Center Houston Texas USA

**Keywords:** atlantoaxial instability, neurosurgery, TB odontoid

## Abstract

**Key Clinical Message:**

Tuberculosis (TB) of the odontoid process is a rare but potentially, a debilitating condition. Surgical intervention, in the form of stabilizing the spine and decompressing the spinal cord, offers a tailored approach to managing this condition effectively and improving prognosis.

**Abstract:**

Odontoid process tuberculosis (TB) is a rare condition that can cause spinal instability and neurological complications. Diagnosis of odontoid process TB is difficult and requires a combination of clinical, radiographic, and histopathological examinations. This report describes the treatment of a 46‐year‐old female with quadriparesis and intermittent fever. Radiological findings showed TB of the odontoid process with atlanto‐axial dislocation causing compressive myelopathy. She underwent C1–C3 decompressive laminectomy and stabilization from C1 to C5. GeneXpert for TB was positive and she was started on anti‐tuberculous medications. She regained power gradually and at 1 year follow‐up she was ambulant without any support. The C1–C5 lateral mass screw and C1–C3 decompressive laminectomy approach, as highlighted in this case, offers an effective solution, enhancing patient quality of life, and preventing disease progression.

## Graphical abstract

Sagittal MRI demonstrating destructive lesion in the odontoid process with pannus formation and compression. 1B: Axial MRI demonstrating the destruction of the anterior elements of C2 with pannus and involvement of the ligamentous complex.

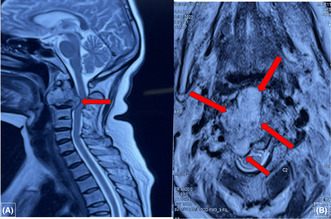

## 1 INTRODUCTION

Tuberculosis (TB) remains one of the leading infectious diseases endemic not only in the low‐income countries but also emerging in developed countries following global migration, HIV epidemic, increased elderly population, immunosuppressive conditions, and drug resistance.^1^, ^2^, ^3^ Spinal TB is the most common form of musculoskeletal TB, accounting for 1% of total TB cases, whereas Cervical Tubercular Disease (CTB) accounts for 3%–5% of all spinal diseases.^2^, ^4^ Craniovertebral junction TB (CVJ TB) or “upper cervical” TB constitutes C1 and C2 vertebrae (Atlantoaxial AATB) and is a rare form of CTB that includes 0.1%–3% of all spinal TB cases.^1^, ^5^ Due to its location, CVJ TB can result in atlantoaxial dislocation, upper cervical spine instability, paravertebral abscess, and neurological deficits.^1^, ^6^ Clinical manifestations may range from non‐specific symptoms including discomfort and neck pain to neurological dysfunction.^1^, ^3^, ^7^

Very few studies have been reported involving the odontoid process as part of CVJ TB.^2^ Due to the inadequacy of literature, the presentation of an ideal approach for diagnosis and treatment options is still debatable. Although non‐complicated early infections can be managed effectively with anti‐tubercular treatment (ATT) and severe neurological deficits often, requiring surgery,^1^ the algorithms are insufficient to treat patients with complexities including significant instability or deformity without neurological deficits.^8^ We report a rare case of CVJ TB with odontoid process involvement resulting in atlantoaxial instability to further highlight the tailored approach in its management.

## 2 CASE REPORT

A 46‐year‐old Indian female presented with complaints of weakness in all four limbs, intermittent fever for the past 15 days and also with a history of weight loss (20 kg) in the past 6 months. There was no past history of any other pre‐existing illness. Routine blood investigations at admission were within normal limits.

**FIGURE 1:**
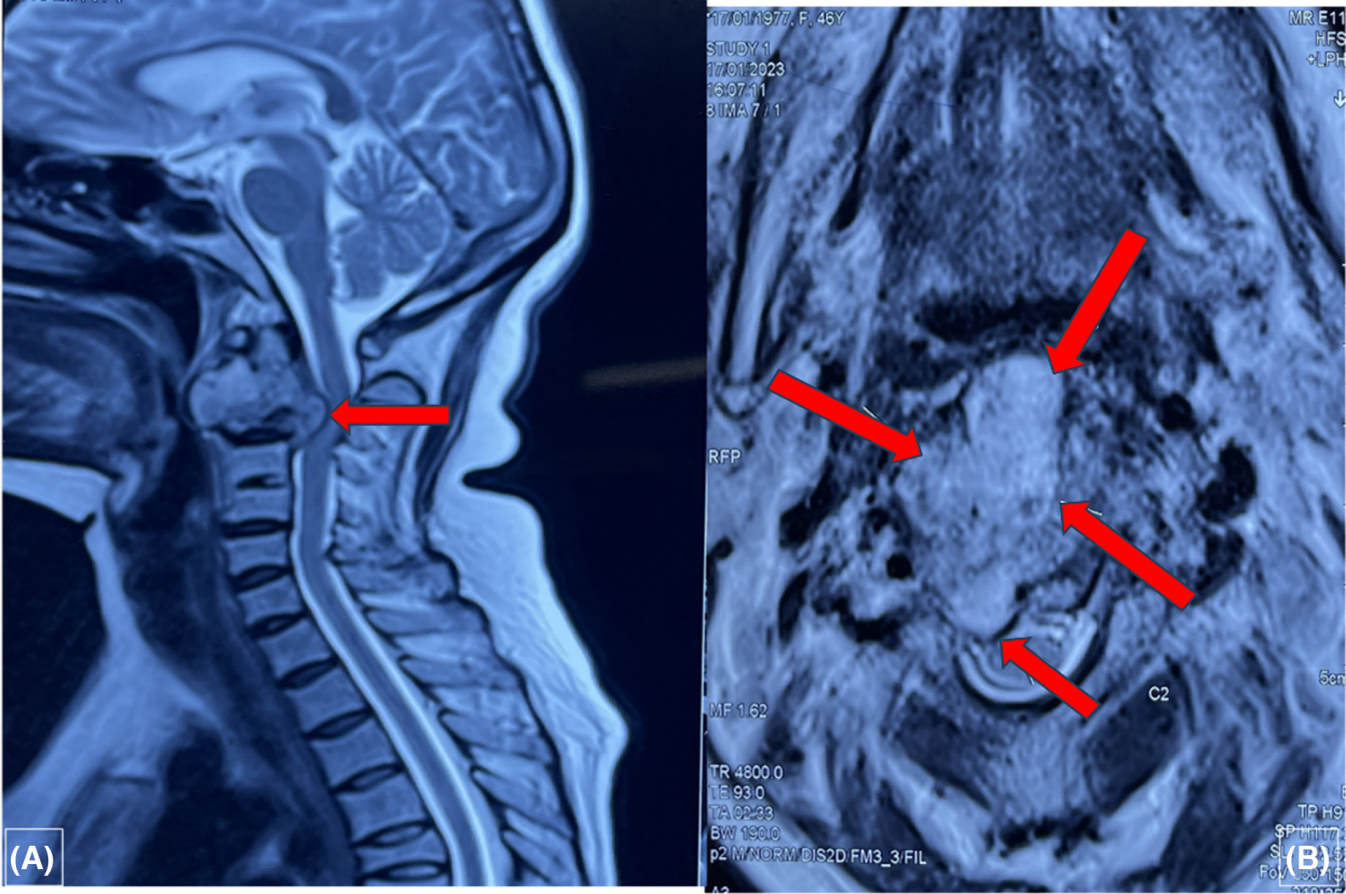
(A) Sagittal magnetic resonance imaging (MRI) demonstrating destructive lesion in the odontoid process with pannus formation and compression. (B) Axial MRI demonstrating the destruction of the anterior elements of C2 with pannus and involvement of the ligamentous complex.

**FIGURE 2:**
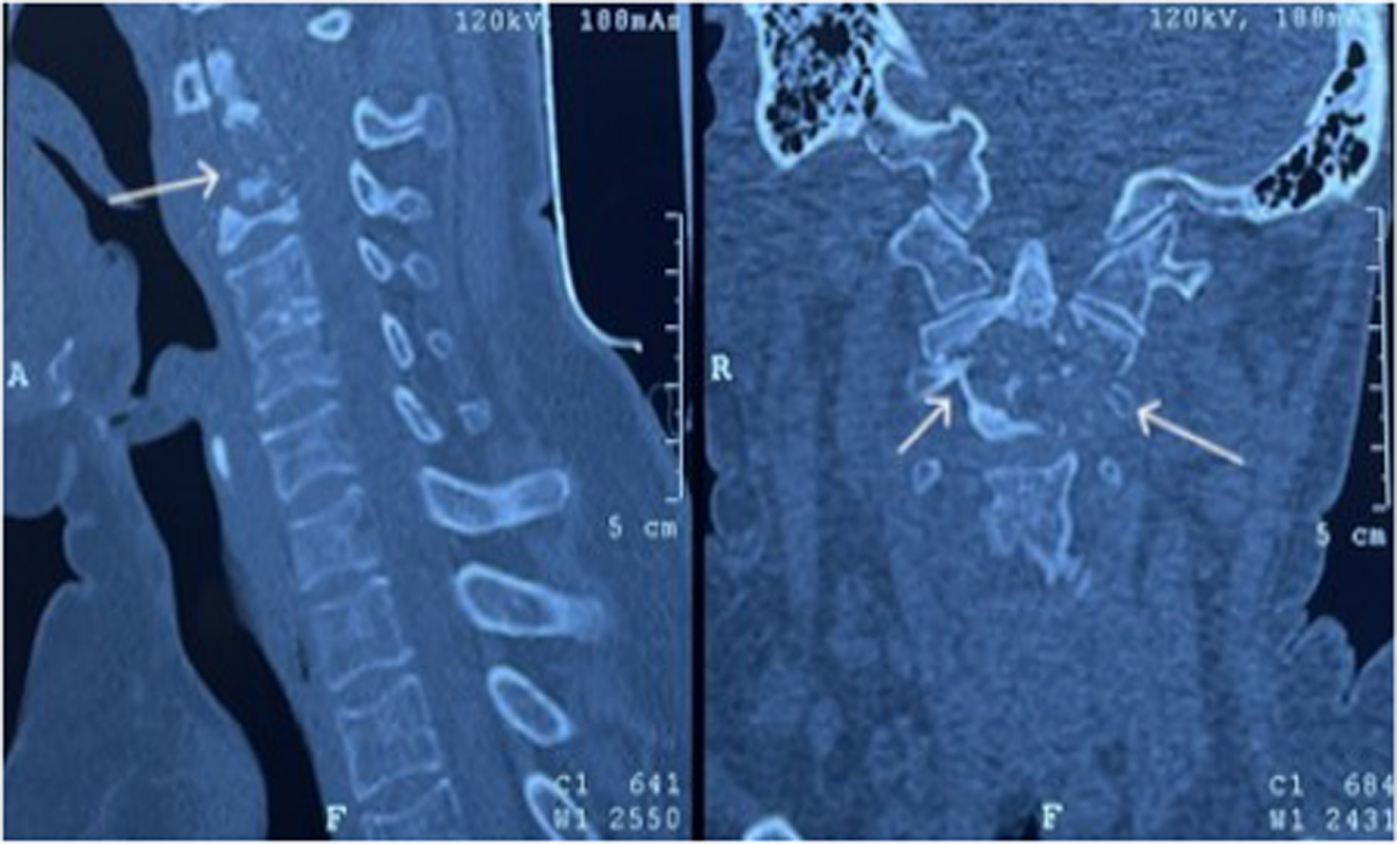
Computed tomography (CT) scan demonstrating the bony destruction at C2.

Computed tomography (CT)–cervical spine was done and showed features of bone destruction, and calcified paraspinal mass with compression, suggestive of tuberculous spondylodiscitis. Further, an MRI of the cervical spine demonstrated a destructive lesion in the odontoid process with pannus formation and compression, and involvement of the ligamentous complex (Figures 1A,B and 2). To investigate other sites of TB, chest x‐ray along with sputum culture for acid‐fast bacilli (AFB) were performed. Chest x‐ray did not show any signs of TB, AFB stain from sputum was negative and culture did not yield *Mycobacterium Tuberculosis* bacteria. An ultrasound scan of the abdomen was performed, which was also negative for any features suggestive of gastrointestinal TB. There was no family history of TB in this patient. She was diagnosed with type 2 diabetes mellitus (T2DM) at the time of present admission.

After obtaining informed consent and anesthetist's fitness, the patient was scheduled for surgery. A C1–C5 lateral mass screw and a C1–C3 decompressive laminectomy and stabilization was performed. It was done under endotracheal tube intubation general anesthesia. The patient was held in a prone position with the head held in Mayfield three‐pin. A midline vertical skin incision was made to expose the subperiosteal muscle. Followed by the dissection of this muscle, the C1–C5 lateral mass was exposed. Under fluoroscopic guidance, lateral mass titanium polyaxial screws were inserted at C1 (3.5 × 28 mm) and C3–C5 (3.5 × 12 mm).

Post that, a C1–C3 decompressive laminectomy was done and rods were placed and fixed. After the posterior decompression and stabilization, the spinal cord was free, and no ventral reconstruction was contemplated. The pannus was evacuated via the posterior approach and sent for analysis. GeneXpert revealed a positivity for TB. She was transfused two units of packed cells and two units of fresh frozen plasma during the procedure. She tolerated the procedure well. As per the Indian extra‐pulmonary TB (INDEX‐TB) guidelines, this patient was started on a 2‐month intensive phase consisting of four drugs (isoniazid, rifampicin, pyrazinamide, and ethambutol), followed by a continuation phase lasting 16 months. She is under regular follow‐up and has not yet completed the full course of treatment. She was also managed with antibiotics, analgesics, gastroprotectives, antidiabetics, supplements, and other supportive measures. Following routine dressing, she was found to be hemodynamically stable, symptomatically improved and hence discharged. At 1‐year follow‐up, the patient's symptoms, such as neck pain, radicular pain, and neurological deficits, improved significantly.

## 3 DISCUSSION

TB is a chronic infectious disease caused by the *M*. *tuberculosis* bacterium. It primarily affects the lungs, but it can affect other parts of the body as well, including the spine. The dens, or odontoid process, is a small bony projection located at the base of the second cervical vertebra (C2). Odontoid process TB, also known as cervical spine TB, is a rare but potentially debilitating condition that can cause spinal instability and neurological complications.^9^

Although the exact incidence of TB of the odontoid process is not known, it is thought to account for less than 1% of all spinal TB cases.^1^ This condition is common among people who live in TB‐endemic areas and in those suffering from immunodeficiency states.^10^ Due to the condition's rarity and non‐specific symptoms, it is frequently misdiagnosed. The diagnosis of TB of the odontoid process is challenging and frequently necessitates a combination of clinical, radiographic, and histopathological examinations. This condition is typically treated with a combination of ATT and surgical intervention.^8^ However, the best way to manage this condition is still being debated among healthcare professionals and researchers, with some recommending a complete conservative management even in advanced cases while others recommending a surgical approach to prevent progression to adverse neurological outcomes.^7^, ^8^, ^11^, ^12^, ^13^, ^14^

The conservative management mainly incorporated immobilization, and traction followed by an efficient drug regimen to reduce the impact while ensuring stability and prevention of compressive myelopathy.^11^ According to Arun Kumar et al., surgical treatment is usually indicated in cases of neurological defects in addition to atlantoaxial instability,^15^ as seen in this patient. They also suggested odontoidectomy and anterior decompression, either separately or together. Qureshi et al, on the other hand, were in favor of posterior stabilization rather than anterior stabilization with plate/rod or screws, with a strong recommendation for lateral mass screws.^8^ Trans articular screw fixation was recommended by Bapat et al. for atlantoaxial instability treatment,^10^ but it is typically difficult as a standalone procedure in cases with C1 involvement.^8^ Golwala et al. also reported on the use of a tricortical iliac crest graft to reconstruct the odontoid process and improve stabilization and incorporation.^16^

With the implementation of the Revised National Tuberculosis Control Programme (RNTCP), India has developed comprehensive guidelines for the diagnosis and treatment of pulmonary TB.^17^ However, management of extra‐pulmonary TB (EPTB) under the program continues to be a challenge. The burden of EPTB is high, ranging from 15% to 20% of all the TB cases in HIV‐negative patients, while in HIV‐positive people it accounts for 40%–50% of new TB cases.^18^

In the present case, a 46‐year‐old female patient was diagnosed with TB of the odontoid process and was treated with surgical intervention followed by a combination of anti‐tubercular drugs. Following the recommendation of Quereshi et al. for the management of patients with Grade 3 and 4 disabilities, the following condition was treated surgically.^8^ The surgical approach included a C1–C5 lateral mass screw and a C1–C3 decompressive laminectomy. A summary of previous studies is presented in Table 1.

**TABLE 1:** Table enlisting published cases of TB involving the odontoid process.

Authors	Location	Age (years)/sex	Symptoms	Treatment	Outcomes
Gupta et al.^19^	India	22 M	Neck & back pain, neck swelling	Rifampicin, isoniazid, pyrazinamide, and ethambutol	Swelling decreased after 10 months, symptom‐free at 18 months
Araneta et al.^20^	Philippines	20 M	Neck pain, weight loss, malaise, and cough	Quadruple antituberculous therapy, antibiotics	Improved neck pain (10/10 to 4/10)
Weber et al.^21^	Germany	17 F	Fever, malaise, weight loss, neck & back pain	Isoniazid, rifampin, and ethambutol	Complete recovery
Diom et al.^22^	Senegal	7 M	Neck swelling, odynophagia, and night sweats	Antibiotics, antitubercular polychemotherapy	Improved after 5 months of treatment
Dhammi et al.^23^	India	45 F	Right hemiplegia	INH, rifampicin, ethambutol, and pyrazinamide	Motor power recovered in 6 months, stable spine at 22 months
		23 M	Neck pain and restriction	Anti‐tubercular therapy	Complete neural recovery at 48 months
		20 F	Neck swelling	Four‐drug anti‐tubercular therapy	After 6 weeks, neural recovery achieved; C1, C2 instability required surgery
Kumar et al.^24^	India	26 F	Neck pain, limb weakness	Neck immobilization, steroids, and antitubercular drugs.	Improved mobility and resolved the abscess
Sengupta et al.^25^	United Kingdom	35 F	Neck pain, stiffness, weight loss, and night sweats for 4 months	Anti‐tubercular drugs, C3, C4 laminectomy, tricortical iliac crest bone graft and locking plate were used	Complications but good fusion, tubercular control
Ebadi et al.^26^	Iran	41 F	Neck and suboccipital pain	Four drug antimycobacterial regimen	Asymptomatic after 18 months
Ding et al.^27^	China	63 M	Neck and thoracic pain	C2–C4 laminectomy, anti‐tuberculous drugs	Full neurological recovery at 1 year
Current case	India	46F	Weakness in all limbs, intermittent fever and weight loss	C1–C3 decompressive laminectomy, antitubercular therapy	She is under follow‐up currently and has not completed the full course of treatment

Abbreviations: C, cervical vertebrae; F, female; M, male; MRI, magnetic resonance imaging; TB, tuberculosis.

The C1–C3 decompressive laminectomy is a surgical technique that involves removal of the affected vertebral bodies' lamina, or roof, to relieve pressure on the spinal cord and nerve roots, thereby effectively treating cervical myelopathy.^28^ This procedure was thus used to treat spinal cord compression caused by TB of the odontoid process. In this patient, a combination of C1–C5 lateral mass screws and C1–C3 decompressive laminectomy proved effective in the management of TB of the odontoid process.

## 4 CONCLUSIONS

TB of the odontoid process is a rare but serious condition with the potential for significant morbidity. The C1–C5 lateral mass screw and C1–C3 decompressive laminectomy approach, as highlighted in this case, offers an effective solution, enhancing patient quality of life and preventing disease progression. Further research is essential to validate the safety and efficacy of this surgical method in larger patient cohorts and to advance our understanding of its epidemiology, diagnosis, and management.

## AUTHOR CONTRIBUTIONS

**Shyam Duvuru:** Conceptualization; data curation; funding acquisition; investigation; methodology; project administration. **Vivek Sanker:** Methodology; project administration; resources; software; supervision; validation; visualization; writing – original draft; writing – review and editing. **Tirth Dave:** Resources; software; validation; visualization; writing – original draft; writing – review and editing. **Pratik Agarwal:** Resources; software; supervision; validation; visualization; writing – original draft; writing – review and editing. **Naureen Syed:** Project administration; validation; visualization; writing – original draft; writing – review and editing.

## ETHICS STATEMENT

The ethical approval was not required for the case report as per the country's guidelines.

## CONSENT

Written informed consent was obtained from the patient to publish this report.

## Data Availability

The data that support the findings of this study are available on request from the corresponding author. The data are not publicly available due to privacy or ethical restrictions.
